# Blood pressure monitoring following individual carpules of anesthetic utilizing computer-controlled anesthetic delivery

**DOI:** 10.1038/s41405-020-00049-y

**Published:** 2020-11-02

**Authors:** Kristi M. Soileau, Adam E. DeGenova, Qingzhao Yu

**Affiliations:** 1 Gratis Faculty, Practice Limited to Periodontics and Louisiana State University Health School of Dentistry, New Orleans, LA USA; 2 Healthcare Administration, New Orleans, LA USA; 3 Louisiana State University Health Sciences Center, New Orleans, LA USA

**Keywords:** Dentistry, Dental treatments

## Abstract

To date, no study has evaluated blood pressure following administration of each carpule given for dental procedures using a computerized dental anesthesia system. Blood pressures taken prior to performing invasive periodontal procedures were compared with those readings measured following delivery of each of up to three consecutive carpules of Marcaine or Xylocaine in varying order. Pressure differences were also adjusted for age, sex, race, and whether a prescribed anxiolytic was taken beforehand. Neither systolic nor diastolic blood pressures changed significantly as compared to initial blood pressure readings. However, compared with Whites, Hispanics, and Middle Easterners, Blacks had significantly higher systolic pressure at the third carpule delivery, the cause being unknown. Blood pressure in patients being anesthetized for root planing and various periodontal surgical procedures will not increase significantly when administering up to three carpules, whether Marcaine or Xylocaine, in varying order, using controlled flow dental anesthesia, and this method may be preferable to syringes in managing dental procedural stress.

## Introduction

Anxiety is a barrier to dental attendance.^1^ It is estimated that approximately half of the people in the United States fear dentistry to some degree, of which ~30 million are termed “phobic,” such that they avoid dental care altogether.^2^ Much of this fear stems from the sight of the anesthetic apparatus and the sensation of the operating dental handpiece, these having been listed as the two most fear-eliciting stimuli.^3^

The injection of local anesthesia can inflict discomfort, which may elicit cardiovascular changes such as hypertension or tachycardia.^4^ For many patients undergoing dental procedures, pain control is essential, particularly in those with cardiovascular disease. Pain and other stressors can result in a dramatic endogenous release of epinephrine and norepinephrine, which can affect heart disease deleteriously; therefore, the importance of good local anesthetic technique cannot be over emphasized.^5^

Epinephrine is widely used as an additive in local anesthetics, most typically in concentrations of 1:100,000 in 2% Xylocaine. This improves the depth and duration of the anesthesia, and reduces bleeding in the operative field. Epinephrine counteracts the anesthetic’s localized vasodilator effects in subcutaneous and submucosal vessels, thereby reducing the risk of anesthetic toxicity while decreasing the rate of systemic absorption from the site of the injection.^6^ Quite commonly, a longer lasting anesthetic with 1:200,000 epinephrine in 0.05% Marcaine is employed in root planing and surgical procedures, depending on patient circumstance.

For certain patients, the stress and anxiety associated with even simple, noninvasive dental visits is enough to increase blood pressure. The common medical condition called white coat hypertension (or white coat syndrome) is important for dental professionals to acknowledge, and routine monitoring of patients in this situation can help prevent possible negative effects associated with this situation. Carotid atherosclerosis is considerably greater in patients with white coat hypertension when compared to those without; therefore, it has been stated that white coat hypertension is a risk factor for heart attack, heart failure, and stroke.^7^

New guidelines from the American Heart Association and the American College of Cardiology in December of 2017 have redefined hypertensive thresholds, with readings of 130/80 now considered to be elevated, or definable as hypertension stage one.^8^ Optimal blood pressure is <120 systolic and <80 diastolic, and roughly one in three adults in the United States is diagnosed as having hypertension.^7^

Hypertension is a highly prevalent cardiovascular disease, which affects over 1 billion people worldwide.^9^ Monitoring of vital signs can help minimize potentially serious adverse drug events.^10^ The dentist’s role in screening for hypertension as part of a comprehensive dental care evaluation is very important.^11^ Risks for hypertension associated with epinephrine include increased acute hypertensive or hypotensive episodes, angina pectoris, arrhythmias, and myocardial infarction.^12^

The perception of pain during administration of local anesthesia can contribute to the stress of the dental visit. Discomfort can be attributed to tissue puncture, fluid pressure, and the flow rate of the drug administered,^13^ while other factors that may influence discomfort are fluid temperature and management of the needle upon full penetration of the tissue. Many patients associate injections of local anesthetic with pain, despite its benefits in treatment.

The most widely used method of injection continues to be the metallic aspirating cartridge syringe system where flow rate and pressure are operator dependent, making it rather difficult to manage injection discomfort. Alternatively, a computerized dental anesthesia delivery system more precisely controls flow rate and fluid pressure by the use of a microprocessor and electronically controlled motor.^14^

The computer-controlled delivery device accommodates a conventional local anesthetic cartridge that is linked by tubing to a disposable pen-like plastic dispenser with an attached luer-lok needle.^13^ It is operated by means of a small rheostat placed on the floor and which delivers local anesthetic at controllable pressure and volume rates.^15^

Several studies have shown this device to be well-received by patients. Twenty subjects undergoing scaling and root planing reported less pain with computer-controlled injections versus that from a traditional syringe as indicated by a visual analog scale.^16^ Additionally, a split-mouth study in which 50 subjects used subjective scales to describe pain experience found the computer to be on average two to three times less painful than manual injection.^14^

Another study in Turkey analyzed 40 patients between the ages of 18 and 30 over two separate dental visits, each subject randomly having traditional delivery of anesthetic at one appointment versus computer-controlled delivery at a subsequent appointment. The subjects then assigned a pain rating score to the two methods, and indicated they would prefer the computer delivery system for future injections.^17^

Blood pressure monitoring, likely a more reliable indicator of the physiologic effects of stress in response to injection than the aforementioned subjective response analyses, has been employed in several syringe-employed anesthetic studies. One study comparing blood pressure readings while using a syringe to administer differing concentrations of epinephrine found no significant variances among the three groups which were studied.^18^ Another study found blood pressure increased during injections by syringe in four groups of patients undergoing extraction, regardless of their ASA status.^19^ Furthermore, Japanese researchers found with the use of a Holter monitor, dental surgery using local anesthesia caused a significant increase in systolic blood pressure, but not in the diastolic, and that the increase in pressure was greater in middle aged and older patients.^20^

In another study where blood pressures were monitored with and without administration of local anesthetic using a syringe, a transient blood pressure increase was found followed by a decrease shortly after removal of the needle from the mouth.^21^ Additionally, in a study of four groups of patients ranging from normotensive to severely hypertensive and who were undergoing tooth extraction with local anesthetic, again utilizing syringes, it was found that blood pressure increased in all groups, with the greatest increase found in those already diagnosed as hypertensive.^19^

It appears to date, however, that no prior study has been conducted measuring physiologic response via blood pressure monitoring with the use of the computerized controlled flow anesthesia delivery system, nor has there been any dental anesthetic blood pressure study performed to assess blood pressure following administration of each carpule of anesthetic administered for dental procedures. Furthermore, this study separated for differences in age, sex, race and effects of the use of an anxiolytic. Seeing as how anesthetic can cause anxiety, and because patient monitoring is important in measuring such stress, this study was undertaken to evaluate blood pressure readings in up to 149 patients at baseline, as well as after each carpule administered, varying second and third carpules between Xylocaine and Marcaine, using a computerized controlled-delivery anesthesia system.

The null hypothesis in this study was that blood pressure (both systolic and diastolic) will not increase significantly during administration of anesthesia as compared to the patients’ initial readings when using the computer-controlled delivery system.

## Materials and methods

IRB exempt status was obtained due to the blind nature of this study. Blood pressure was measured at baseline and again following delivery of each of three consecutively administered carpules of dental anesthetic in 149 periodontal patients previously scheduled for either root planing or periodontal surgical procedures which included cosmetic and functional crown lengthening, osseous surgery, and soft tissue grafting. Blood pressure readings were taken at baseline and immediately following the emptying of each consecutive carpule. The blood pressure monitor used was the Mindray Datascope Trio Patient Monitor, which also monitored pulse oximetry and electrocardiogram activity throughout procedures. The anesthetic delivery system employed, whereby carpule administration occurs via a flow rate that is controlled by use of a microprocessor and electric motor, was used in all patients. No adverse effects were noted during any of the procedures, while a variety of injection types were employed, all with 27 gauge needles, with approximately half of the patients receiving palatal injections. Vasoconstriction was utilized due to the need for increased anesthesia duration.

Patients were previously prescribed 0.5 mg of Alprazolam, to be taken 1 h prior to the scheduled procedure (with 39 patients opting not to take the medication, and 4 opting to use their own already-prescribed antianxiety medication). The first carpule given to all patients was exclusively Xylocaine 2% with 1:100,000 epinephrine. In 116 of these patients, the second carpule given was another Xylocaine 2% with 1:100,000 epinephrine, while in 33 of these patients the second was 0.5% Marcaine with 1:200,000 epinephrine. A third carpule was then given in 116 patients, wherein 99 patients had another carpule of 2% Xylocaine with 1:100,000 epinephrine, while 17 had a carpule of 0.5% Marcaine with 1:200,000 epinephrine only in those patients whose first two carpules had been exclusively Xylocaine. Of these, 34 patients had not taken any antianxiety medication, and 4 opted to take their own already-prescribed medication.

A one-sided paired *t*-test to compare blood pressure following administration of each carpule separately with the initial blood pressure reading was utilized. Because there was a maximum of three carpules in total for each subject, separate hypothetical comparisons were conducted for carpules 1–3 where applicable. The Bonferroni correction method was employed to control the familywise type one error rate at a 0.05 level for multiple comparisons. Linear regressions were also adopted to compare differences controlling for age, sex, race, and whether patients had antianxiety medication or not before the procedure.

## Results

### Comparison of the change in blood pressure while varying the second type of anesthetic carpule when the first carpule was Xylocaine

#### Second carpule type relative to change in blood pressure (sample size 149)

##### Systolic pressure

**Fig. 1:**
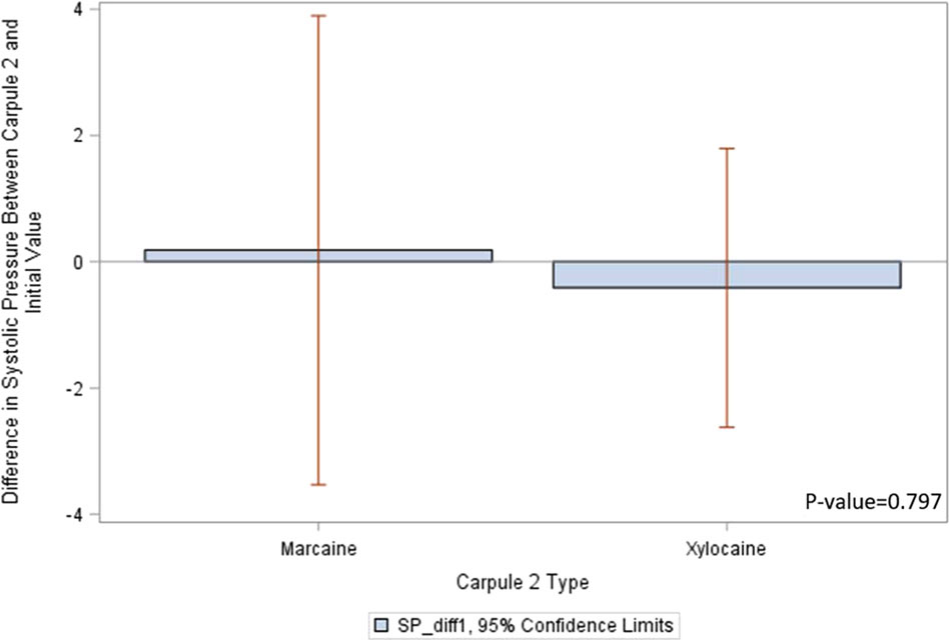
Systolic pressure differences with Carpule 2. This figure shows systolic pressure difference Marcaine and Xylocaine with 95% confidence interval.

Figure 1 provides a descriptive analysis and shows *t*-test results for the relationship between the type of second carpule, following a first carpule of Xylocaine, and the difference in systolic pressure from the initial pressure reading. Using Xylocaine as carpule 2, the average change in systolic pressure is positive (lower at initial), while the average change is negative (lower at carpule 2) when using Marcaine as the second carpule. However, the *p* value from a one-way ANOVA test is 0.80, indicating that the relationship between the type of second anesthetic carpule and the change in systolic pressure is not different at a significance level of 0.05.

##### Diastolic pressure

**Fig. 2:**
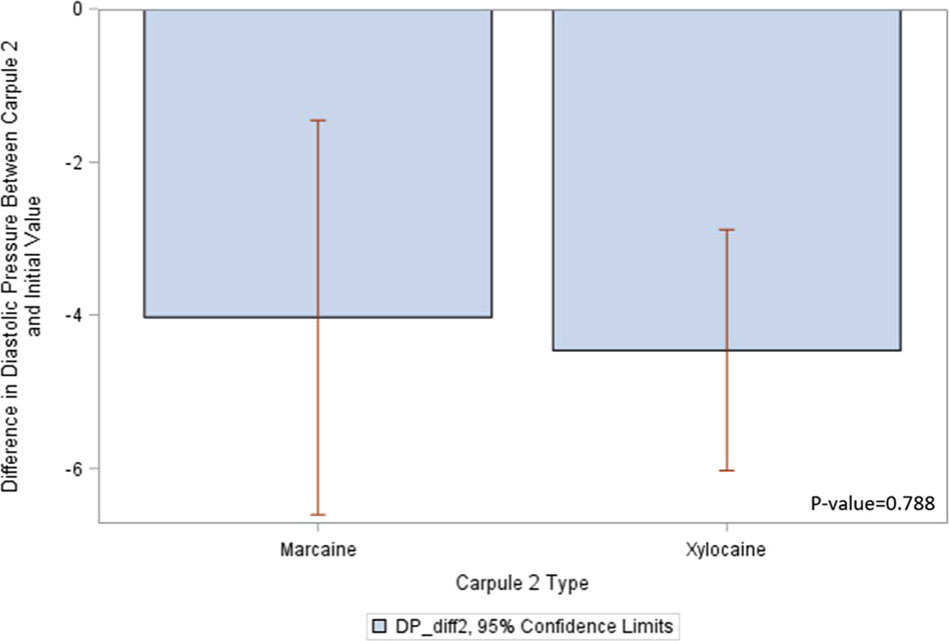
Diastolic pressure differences with Carpule 2. This figure shows diastolic pressure difference between Marcaine and Xylocaine with 95% confidence interval.

Figure 2 provides descriptive analysis and shows *t*-test results. The *p* value of 0.79 indicates that there is no significant relationship between the type of second anesthetic carpule and the change in diastolic pressure.

#### Second carpule type relative to change in blood pressure when adjusting for age, sex, race, and use of anxiolytic

##### Systolic pressure

Table 1 shows the results using a linear regression model, between the type of second carpule and the change in systolic pressure when controlling for other factors. The *p* value of 0.87 indicates that when adjusting for age, sex, race, and anxiolytic, the type of second anesthetic has no significant effect on the change of systolic pressure. In addition, none of these factors significantly relate to change in systolic blood pressure.

**Table 1:** Systolic pressure differences between carpule 2 and baseline when adjusting for age, sex, race, and use of anxiolytic.

Source	Df	Type III SS	Mean square	*F* value	Pr > *F*
Carp2	1	3.368335	3.368335	0.02	0.8753
Age	1	0.010493	0.010493	0.00	0.9930
Sex	1	13.947864	13.947864	0.10	0.7494
Race	4	1130.986006	282.746502	2.08	0.0872
Anxiolytic	3	265.917646	88.639215	0.65	0.5837

##### Diastolic pressure

Table 2 shows the results using a linear regression model. The *p* value of 0.81 indicates that there is no significant relationship between the type of third anesthetic carpule and the change in diastolic pressure when controlling for age, sex, race, and anxiolytic. Also these other factors do not significantly relate with the change of diastolic pressure.

**Table 2:** Diastolic pressure difference between carpule 2 and baseline when adjusting for age, sex, race, and use of anxiolytic.

Source	Df	Type III SS	Mean square	*F* value	Pr > *F*
Carp2	1	3.7096176	3.7096176	0.06	0.8131
Age	1	157.3624900	157.3624900	2.38	0.1251
Sex	1	1.7484312	1.7484312	0.03	0.8710
Race	4	131.1826275	32.7956569	0.50	0.7385
Anxiolytic	3	156.5972030	52.1990677	0.79	0.5015

### Comparison of the change in blood pressure while varying the third type of anesthetic carpule delivery, measured only in those individuals whose first two anesthetic deliveries were both Xylocaine

#### Third carpule type relative to change in blood pressure (sample size 116)

##### Systolic pressure

**Fig. 3:**
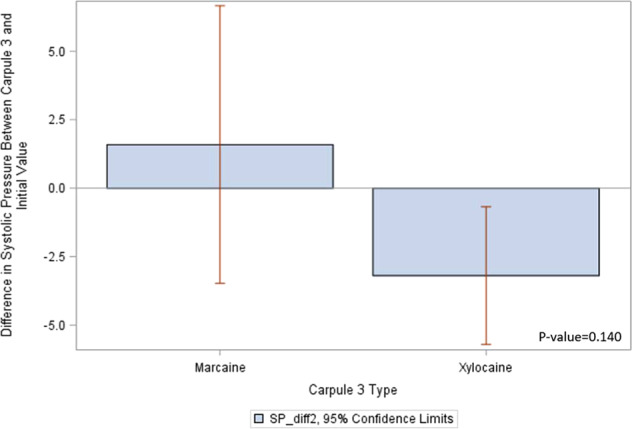
Systolic pressure differences with Carpule 3. This figure shows systolic pressure difference between Marcaine and Xylocaine with 95% confidence interval.

Figure 3 provides a descriptive analysis and shows *t*-test results. The *p* value of 0.14 indicates that there is no significant relationship between the type of the third anesthetic carpule and the change in systolic pressure among individuals whose first two carpules were both Xylocaine.

##### Diastolic pressure

**Fig. 4:**
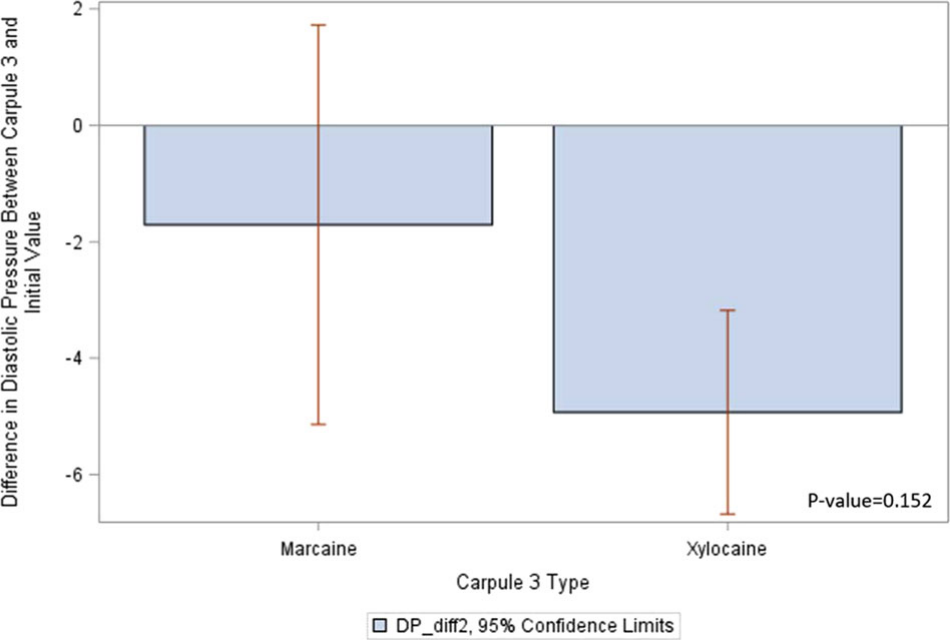
Diastolic pressure differences with Carpule 3. Diastolic pressure difference between Marcaine and Xylocaine with 95% confidence interval.

Figure 4 provides a descriptive analysis and shows *t*-test results. The *p* value of 0.1519 indicates that there is no significant relationship between the type of the third carpule and change in diastolic pressure among individuals whose first two carpules were both Xylocaine.

#### Third carpule type relative to change in blood pressure when adjusting for age, sex, race, and use of anxiolytic

##### Systolic pressure

Table 3 shows the results using a linear regression model, indicating no significant relationship between the type of third anesthetic carpule and the change in systolic pressure when controlling for other factors. However, race was an important predictor for change in systolic pressure. Compared with Whites, Hispanics, and Middle Easterners, Blacks exhibited a significantly larger change in systolic pressure at the third carpule delivery.

**Table 3:** Systolic pressure difference between carpule 3 and baseline when adjusting for age, sex, race and use of anxiolytic.

Source	Df	Type III SS	Mean square	*F* value	Pr > *F*	
Carp3	1	232.596010	232.596010	1.62	0.2055	
Age	1	16.454253	16.454253	0.11	0.7354	
Sex	1	220.300712	220.300712	1.54	0.2178	
Race	4	1543.569443	385.892361	2.69	0.0349	
Anxiolytic	3	354.242973	118.080991	0.82	0.4835	

Parameter		Estimate		Standard error	*t* value	Pr > |*t*|
Intercept		−5.558442339	B	4.05232248	−1.37	0.1731
Carp3 B		4.197873317	B	3.29490690	1.27	0.2055
Age		−0.023502511		0.06935697	−0.34	0.7354
Sex F		2.932335498	B	2.36494289	1.24	0.2178
Race W		5.232498666	B	7.42220356	0.70	0.4824
Race B		8.498361585	B	2.60741703	3.26	0.0015
Race H		1.562324812	B	8.88518094	0.18	0.8608
Race ME		4.193010139	B	8.88903784	0.47	0.6381
Anxiolytic clonazepam		−6.908651789	B	12.24589851	−0.56	0.5738
Anxiolytic N/A		−2.829645521	B	2.61479368	−1.08	0.2817
Anxiolytic diazepam		6.126917051	B	7.37210232	0.83	0.4078

##### Diastolic pressure

Table 4 shows the results using a linear regression model. There is no significant relationship between the type of third anesthetic carpule and the change in diastolic pressure when controlling for age, sex, race, and anxiolytic. Also, none of these factors significantly relate to change in diastolic blood pressure.

**Table 4:** Diastolic pressure difference between carpule 3 and baseline when adjusting for age, sex, race, and use of anxiolytic.

Source	Df	Type III SS	Mean square	*F* value	Pr > *F*
Carp3	1	68.1526132	68.1526132	0.92	0.3391
Age	1	12.7778117	12.7778117	0.17	0.6784
Sex	1	7.2304899	7.2304899	0.10	0.7551
Race	4	206.6564365	51.6641091	0.70	0.5943
Anxiolytic	3	254.3502488	84.7834163	1.15	0.3337

This study showed no difference in adjusting for race except that compared with Whites, Hispanics, and Middle Easterners, Blacks had a significantly larger change in systolic pressure at the third carpule delivery with the computer-controlled-flow anesthesia system. The reason for this within the parameters of this study is unclear. However, generally, it was found that both systolic and diastolic blood pressures did not increase significantly as compared to initial blood pressure readings following administration of three carpules of Marcaine or Xylocaine, in varying order, using the computerized delivery anesthetic system, showing this to be a possible method of reducing stress in anesthesia administration.

This study showed no difference in adjusting for age and sex. Regarding the effects of anxiolytics, and using Alprazolam as a reference group, while some anxiolytic response showed a relative lower (negative coefficient) blood pressure effect, and others showed a relatively higher (positive coefficient) blood pressure effect, none were significant. In other words, no group was significantly different from the group receiving Alprazolam after adjusting for other variables.

## Discussion

Stress during injections can be a deterrent to successful and less eventful patient treatment, especially in cases of hypertension, yet no study to date has monitored blood pressure with controlled flow or following each carpule administered, separating for age, sex, race, and use of anxiolytic.

This is of interest for several reasons. Currently, up to 46% of United States adults are identified as having high blood pressure.^22^ Of significance is the fact that elevations in both systolic and diastolic blood pressures can be used to make a diagnosis of hypertension, and the risk of death from ischemic heart disease and stroke has been found to double with every 20 mm of mercury (systolic) or 10 mm mercury (diastolic) in persons aged 40–89.^23^ Thus, dental professionals must be on the frontlines of prevention of hypertension by evaluating preoperative blood pressure readings, performing risk assessments, and knowing when to refer a patient for medical consultation in a dental setting.^12^ Studies of the prevalence of dental anxiety in the general population show a range of 2.6–20.4%,^24^ and measurement of blood pressure during the stressful moments surrounding injection-giving helps dentists to avoid untoward cardiovascular events. High levels of stress, including that from dental anesthetic, can indeed lead to increases in blood pressure, even if temporary.^25–27^ Anesthetics were selected according to what best suited each individual patient depending on duration of anesthesia and hemostasis needed. The Lidocaine HCl 2% solution with a 1:100,000 epinephrine concentration used in this study provides average pulpal anesthesia for 60 min, with soft tissue anesthesia for ~2.5 h.^28^ The concentration of 0.5% Marcaine with 1:200,000 epinephrine employed yields a duration of anesthesia two to three times longer than that of the Lidocaine, in many patients lasting up to 7 h.^29^ Furthermore, measuring vital signs frequently where multiple injections are performed has been advised as a consideration and prevention of anesthetic emergencies.^28^

The rate at which anesthetic is injected is seemingly very important in preventing stress in dental patients. Slower administration of a carpule over a period of a maximum of 60 s is recommended for optimal reduction of pain during injection.^30^ This computerized local anesthesia delivery system offered several advantages over a conventional syringe, including controlled, slow flow delivery, likely improved tactile sensation with the lightweight plastic handle, and the ability to rotate the needle as it is introduced into tissue, as well as while aspirating. Disadvantages include the fact that the system is costly, rather complex, bulky, and generally consumes more time for administration of anesthetic.

Interest in race and age differences in blood pressure within this study stems from prior variances in these parameters. Data from a National Health and Nutrition Examination Survey showed that between 2015 and 2016, prevalence of hypertension was 29% and increased with age, with 18–39 years old at 7.5% and those over 60 at 63.1%. Hypertensive prevalence was higher in non-Hispanic blacks at 40.3% than non-Hispanic whites at 27.8%, non-Hispanic Asians at 25.0% or Hispanics at 27.8% in adults. However, hypertension control was higher among the non-Hispanic whites at 50.8% then non-Hispanic blacks at 44.6% or non-Hispanic Asians at 37.4%.^31^ This current study showed that following each of up to three carpules given, no significant increase in blood pressure, except with systolic pressure in Blacks when the third carpule was administered. The cause of this is uncertain. Because hypertension affects ~75 million United States residents,^32^ as well as the fact that the administration of local anesthesia containing epinephrine can affect the cardiovascular system, gentle anesthesia management, and proper monitoring of patients receiving injections is crucial.^5^

Patient variables that were not controlled in this study include the possibility that patients had variances in presurgical meal content, or other drugs which may have affected blood pressure responses,^12,25,33^ possibly exacerbating or compounding the effects of the stress of the injection and the epinephrine contained within the anesthetic.

Also, time between injections and the locale of each injection, as some anatomical locations in the mouth have different tissue resistance,^34^ were not standardized, and therefore could have affected patient-to-patient comparisons. It is conceivable that implementing a future study that controls these factors more astutely and which would employ a larger patient population with each individual factor would likely create greater reliability of the results found herein.
